# Reinforcement Learning Environment for Advanced Vehicular Ad Hoc Networks Communication Systems

**DOI:** 10.3390/s22134732

**Published:** 2022-06-23

**Authors:** Lincoln Herbert Teixeira, Árpád Huszák

**Affiliations:** Department of Networked Systems and Services, Faculty of Electrical Engineering and Informatics, Budapest University of Technology and Economics, Műegyetem rkp. 3, H-1111 Budapest, Hungary; huszak@hit.bme.hu

**Keywords:** advanced vehicular ad-hoc network, routing network, reinforcement learning

## Abstract

Ad hoc vehicular networks have been identified as a suitable technology for intelligent communication amongst smart city stakeholders as the intelligent transportation system has progressed. However, in a highly mobile area, the growing usage of wireless technologies creates a challenging context. To increase communication reliability in this environment, it is necessary to use intelligent tools to solve the routing problem to create a more stable communication system. Reinforcement Learning (RL) is an excellent tool to solve this problem. We propose creating a complex objective space with geo-positioning information of vehicles, propagation signal strength, and environmental path loss with obstacles (city map, with buildings) to train our model and get the best route based on route stability and hop number. The obtained results show significant improvement in the routes’ strength compared with traditional communication protocols and even with other RL tools when only one parameter is used for decision making.

## 1. Introduction

A vehicular ad-hoc network (VANET) allows cars and roadside devices to connect with one another [1]. Even if they have no prior knowledge of other vehicles in the region, automobiles are viewed as nodes in a self-organizing network for communication reasons [2]. This means that there is no preexisting infrastructure required for this decentralized wireless ad-hoc network. Each node acts as a host as well as a router, delivering and receiving data between nodes. To communicate between devices, dedicated short-range communication radios (DSRC) are employed [3]. DSRC is a wireless communication system based on the IEEE 802.11p standard that allows automobiles to communicate directly at high speeds and with high security without the need for cellular infrastructure. Also, the 5.9 GHz frequency is used by DSRC to allow low-latency information sharing between automobiles.

This architecture is in charge of delivering and receiving safety alerts, as well as maintaining passenger and pedestrian safety [4]. It also improves the flow of traffic and the effectiveness of the traffic management system. An onboard unit (OBU) has several sensors such as a GPS, accelerometer, resource command processor, user interface, and read/write storage for storing data. Over an IEEE 802.11p wireless connection, OBUs are in charge of communicating between surrounding devices [5]. The most difficult component of VANET is managing and routing the data required for optimum connectivity.

The data broadcasted and received by vehicular units in ad-hoc vehicular networks comprise information on the telemetry of the related cars. When information is transmitted over the air, it is very sensitive to interference, which can result in a network outage, putting the lives of drivers and anyone near to them in danger. Because there is no large infrastructure involved in a shared wireless medium, there is a high risk of car-to-car communication failure. In multi-hop communication, nodes or vehicular units function as hosts and routers, forwarding and receiving data from other nodes [6].

As a result, nodes are chosen based on their connectivity, so the routing algorithm selected is a sensitive issue that must be handled carefully in this type of system. Routing protocols developed for legacy networks may not adequately serve vehicular networks in the near future. Therefore, new alternatives must be developed to solve the routing problem in these complex networks.

We provide techniques for improving the performance of the methodology we have developed, such as:

(i) Choosing the best path based on signal strength between adjacent nodes and avoiding hosts with high Path Loss to avoid retransmissions;
(ii) Using a reward feature that assesses the path loss between hosts, choose the route with the longest lifespan;
(iii) Analyzing performance parameters like route lifetime, number of reconnects, and hop counts.

The following are the article’s primary contributions:

- We improve the Reinforcement Learning environment where an agent can learn to find the path with the most extended lifetime. A procedure determines the chosen path based on signal power between the nodes, and as a reward, the path’s lifespan is connected.
- The impacts of existing procedures were compared to the novel paradigm suggested in this work utilizing computer-based simulation tools.
- And, we compare the results with two legacy routing algorithms, to prove the efficiency of the proposed work.

The following is how the remainder of this research paper is organized: The Section 2 looks into the related work. The environment model is discussed in Section 3. Then, in Section 4, the outcomes and discussion are defined. The conclusion in Section 5 brings the paper to a close.

## 2. Related Work

As related work, we will address the two traditional routing protocols widely used in ad-hoc networks, DSR and DSDV. These protocols were used in the comparative performance of the planned method. We will also address solutions related to routing path lifetime where current machine learning research is applied in VANET. Lastly, the two Reinforcement Learning algorithms used in our proposed approach were PPO and A2C.

VANETs (vehicular ad hoc networks) have become a prominent research topic in recent years. VANETs are confronted with new development patterns as new technologies arise. Advanced VANETs, which integrate regular VANETs with these upcoming technologies, can increase transportation safety and efficiency while also improving automobile owner experiences. Advanced VANETs, on the other hand, have additional obstacles. To overcome these, new architectures, procedures, and protocols must be devised [7]. This work proposes a new methodology to find routes in this dynamic environment, so we believe that this work is part of the new approach called Advanced Vehicular Networks.

Owing to stochastic node movements, interference, multipath propagation, and path loss, wireless ad-hoc mobile networks lack a consistent topology due to the absence of physical links between nodes. Many routing protocols have been suggested and are continuously being researched in order to reduce the possibility of communication failures with this technology.

VANETs are currently confronting new development trends as a result of the advent of new technologies such as 5G, cloud/fog computing, blockchain, and machine learning [8,9]. Advanced VANETs, which combine standard VANETs with these future technologies, have the potential to boost efficiency dramatically. Advanced VANETs, on the other hand, have new obstacles. To overcome them, new architectures, procedures, and protocols must be developed, as proposed by the journal [10].

### Reinforcement Learning

Reinforcement learning (RL) is the process of determining what actions to take to enhance a quantitative reward value. The learner is not taught which actions to perform; rather, he is encouraged to try them all and see which ones provide the greatest outcomes. Actions can have an influence not only on the immediate, but also on a problem, a class of problem-solving techniques that successfully act on the problem, and the field that analyzes the problem and its answers, under the most intriguing and difficult situations [11].

Reinforcement learning is distinct from supervised learning, which is the focus of the majority of contemporary machine learning research. Learning from a set of labeled examples given by an expert external supervisor is known as supervised learning. Each example is a description of a scenario followed by a specification—the label—of the proper reaction the system should take in response to that condition, which is frequently to identify a category to which the situation belongs. The goal of this sort of learning is for the system to be able to generalize its responses to situations outside of the training set. While this is an important sort of learning, it falls short of what is required for learning through interaction. It is difficult to conceive of examples of desired conduct that are both correct and indicative of all the scenarios in which the agent must respond to interactive concerns. An agent must be able to learn from its own experience in an uncharted territory where learning would be most helpful [12].

RL is also separate from unsupervised learning, which is focused on detecting structure in vast volumes of unlabeled data, as defined by machine learning specialists. The terms supervised and unsupervised learning appear to categorize machine learning paradigms entirely, but they do not. While it is easy to mistake reinforcement learning for unsupervised learning, rather than discovering a hidden structure, the goal of reinforcement learning is to maximize a cumulative reward. While studying the structure of an agent’s experience can help with reinforcement learning, it doesn’t address the problem of maximizing a reward value.

**Figure 1:**
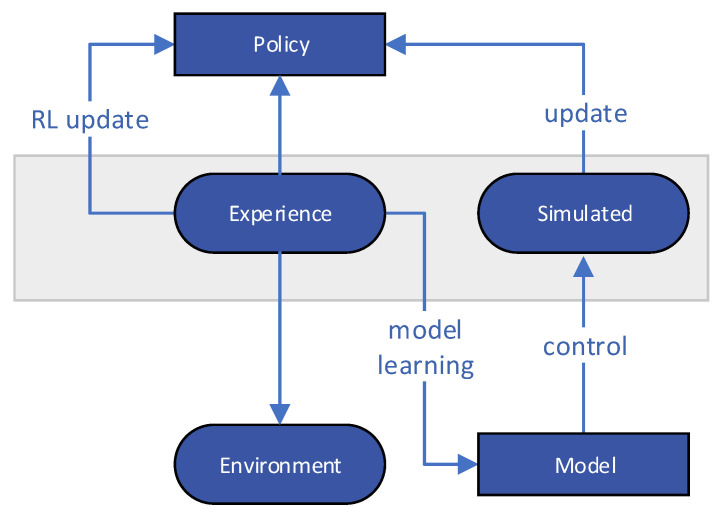
Reinforcement Learning (RL) flowchart.

The trade-off between exploration and exploitation is one of the issues that occur in reinforcement learning but not in other types of learning. To obtain a large number of rewards, a reinforcement learning agent must select activities that it has previously done and found to be useful in terms of reward provision. He must, however, do actions he has never performed before in order to uncover such acts. In order to receive a reward, the agent must not only examine what it has already experienced but also discover how to make better future action options. The issue is that neither exploration nor exploitation can be carried out merely for the purpose of achieving success. Before favoring the actions that appear to be the most successful, the agent must test a number of them. To get a reliable estimate of an action’s anticipated reward, it must be tried multiple times on a stochastic task, as seen in the flowchart in Figure 1. Reinforcement learning also has the advantage of being able to handle any issue involving a goal-directed agent interacting with an unknown environment.

Reinforcement learning begins with a fully functional, interactive, goal-seeking agent. All reinforcement learning agents have specified goals, are able to monitor parts of their surroundings, and may influence their environments by taking actions [13]. Furthermore, it is customary to anticipate that the agent will have to function in an environment with a great deal of uncertainty from the start.

One of the most exciting aspects of today’s reinforcement learning is how successfully integrates with different technological and scientific domains. Reinforcement learning is part of a long-standing trend in AI and machine learning to combine statistics, optimization, and other mathematical subjects. The ability of some reinforcement learning algorithms to train using parameterized approximates, for example, tackles a long-standing problem in operations research and control theory: dimensionality. Reinforcement learning, in particular, has had a fruitful collaboration with psychology and neuroscience, with considerable benefits for both sides. Many of the best reinforcement learning algorithms were influenced by biological learning systems. Reinforcement learning is the closest sort of machine learning to the kind of learning that humans and other animals do.

Therefore RL has been recognized as one of the most effective optimization tools in solving complex problems. Existing RL-based systems, on the other hand, suffer from sluggish convergence for optimum communication due to the improper design of RL elements (i.e., reward and state) for complicated traffic dynamics.

Meanwhile, most optimization approaches assume that the network communication environment’s phase length is constant to simplify RL modeling, which severely limits the RL’s ability to seek up network route management policies with a reduced average number of hops and better communication time stability [14].

In this paper, we assess and examine the efficacy of two legacy VANET routing algorithms, DSR and DSDV, and compared them with our suggested RL-based methodology. As this is an ad-hoc type of communication that will be widely used in vehicular networks in the future, routing algorithms for ad-hoc vehicular networks should be improved when traffic congestion worsens. A traffic control network failure of even a millisecond can be devastating [15].

As a consequence, we present an RL-based method for determining the intensity and length of the communication range between vehicles in order to optimize communication routing. Inspired by the prior work approach used in the transportation business, we established a new concept called intensity, which ensures that our incentive design and state representation accurately reflect the condition of vehicles [16]. Our method allows us to change the communication phase between all hosts involved in the route to adapt to changing traffic conditions while taking into account the coordination of nearby vehicles, the signal strength, noise, and interference of the route loss. Comprehensive experimental findings in artificial and real-world traffic scenarios reveal that this unique method achieves improved average route active duration time and converges to optimal solutions faster than state-of-the-art RL systems [17]. As a result, this method helps to select the most reliable route rather than the shortest way, as most existing routing protocols do, depending on the position of origin, destination, and intermediate nodes that make up the route for information to travel.

While reinforcement learning has had a significant influence in other fields, we believe that its promise in networking has yet to be completely realized.

Therefore, OpenAI Gym [18] is a research toolkit for reinforcement learning that attempts to bring together the finest features of prior benchmark sets in a software package that is both easy and accessible. It consists of a broad set of jobs (called environments) with a common interface, which will continue to expand over time. The environments are versioned in such a way that when the program is updated, the findings will stay meaningful and reproducible.

We hope that by providing an OpenAI Gym environment, researchers will be encouraged to investigate solutions that apply reinforcement learning in network communication systems.

## 3. Environment Model

Instead of considering only the distance between nodes, this work proposes a routing method that considers the signal strength between nodes, taking into account the physical obstacles that degrade the communication. This interferes with the lifetime of the chosen path, until one or more nodes on the path leave the signal range, making the route invalid. Traditional ad hoc routing systems such as DSDV and DSR [19,20] choose a route depending on the number of hops. Although the selected path contains a limited number of intermediate nodes between the source and the destination, the distance between each pair is usually high. If intermediate nodes with shorter distances between them are selected, the chance of path longevity increases.

In the proposed task offloading structure, the agent manages the route table between vehicles. Vehicles in range signal coverage are considered connected and learn the vehicle states in real-time. The state contains the current position, speed, and id of each vehicle. The environment is formed as follows:

The state-space *S*, consists of the current state of the graph (in terms of a multidimensional output of a pro-based pairwise signal strength model) and a multifaceted observation; The action space *A*, which consists of the next hop of the graph; Transitions between states, governed by the deterministic process-based model and the distance sequence; The reward *r* encourages the lifetime of the path.

Observation space: In order to not violate Markov’s property [13], that is, we do not know where the vehicles are moving, the alternative is to save some observations from the past and use them as a state. After receiving the vehicle states, the agent feeds the position matrix, keeping the last four previous positions and updating the current position. We propose to keep together four successive locations and observe each state; thus, this preprocessing stacks four rows, resulting in the final state space in the column array. That is, it is composed of a matrix that has the combination of all possible communication pairs (links) based on the signal strength between the vehicles. The matrix is exemplified in Equation (1).

(1) $$S=\left\{\begin{array}{c} \left[\begin{array}{ccc} RSSI_{t}\left(id_{0},id_{1}\right) & \ldots & RSSI_{t}\left(id_{0},id_{n}\right)\\ RSSI_{t-1}\left(id_{0},id_{1}\right) & \ldots & RSSI_{t-1}\left(id_{0},id_{n}\right)\\ RSSI_{t-2}\left(id_{0},id_{1}\right) & \ldots & RSSI_{t-2}\left(id_{0},id_{n}\right)\\ RSSI_{t-3}\left(id_{0},id_{1}\right) & \ldots & RSSI_{t-3}\left(id_{0},id_{n}\right) \end{array}\right]\\ \vdots \\ \left[\begin{array}{ccc} RSSI_{t}\left(id_{0},id_{1}\right) & \ldots & RSSI_{t}\left(id_{0},id_{n}\right)\\ D_{t-3}\left(0,1\right) & \ldots & D_{t-3}\left(\left(n-1\right),n\right) \end{array}\right] \end{array}\right\}$$

where RSSI means the Received Signal Strength Indicator between the hosts, the meaning is described in the Table 1. The *t* is the current timestamp, id is the identification number of vehicles in a given area, and the number of columns of the matrix is the combination of the pairs of hosts.

In this work, we consider that less than −70 dBm of RSSI signal, we do not consider that the vehicles are connected.

Action space: In this case, the action space is discrete and determined by the number of cars in a certain place, such as downtown. It is made up of automobiles that can serve as origin, destination, or intermediate nodes along the path.

Reward: There are three zones in which the reward function is specified. The package’s destination is represented by the target. When the chosen *S* state may be part of the route, the viability zone is identified. When the specified state does not exist or cannot be a part of the path (due to the RSSI between the current node and the next-hop being weak), the reward function will get the reward value if it reaches the success zone, that means, the target.

The lightest life route of dynamic graphs will be discovered using reinforcement learning methods. Lifetime, Reset, Observation, and Step are the algorithm’s four primary functions. The Lifetime function determines how long the specified path will take to complete. When the route loops, the Reset function is executed. The RSSI is calculated using the Cartesian location of each vehicle, and the current signal value, as well as the previous four readings, are stored between the vehicles via the Observation function. The step function is the algorithm’s fundamental function, in which reinforcement learning algorithms learn to seek the largest reward, i.e., a prolonged lifespan of the chosen path, depending on the observation space after each action. Four different test conditions are available for the Step function. The first is if there is a connection between the present location and the action’s next node. Proceed to the next tests; otherwise, the algorithm will have to choose another node to join the route. To eliminate loops, we require the actioninpath test condition; if the selected action is already part of the path, it signifies that a certain node has already been picked to be part of the path to end a loop. This causes the route to becoming stuck in a loop, which is undesirable in any routing scheme. It is for this reason that the Reset function is used.

Following the first condition, it will be determined whether the selected action is equivalent to the goal; if it is, the path has arrived at its final destination, the lifetime of that path can be calculated and the algorithm for that path can be completed. This is an appropriate action that can be selected if the chosen action is not yet part of the route. The payoff, on the other hand, is still zero, and the process isn’t complete. One more node that might be part of the route path was discovered. The Observation function returns the four standard variables of the OpenAI Gym environment at the end of the condition testing (obs, reward, done, info).

The NetworkX tool is a package of the Python programming language, which creates and manipulates the dynamic structure of complex networks [21]. This tool was used to build the connection of a weighted directed graph between vehicles based on the RSSI signal. The two main functions, H.has_edge, are to check if the link between the current property and the next node is still active, and H.has_path to check how long the route remains active, making it possible to calculate the route lifetime.

RSSI signal: The RSSI is determined by the Radio Tracer multipath engine, which tracks rays for multipath radio wave propagation. The core ray tracing engine is written in the Python C++ extension, and we use a Python package. The route loss calculator is also included in the package. The main engine reads the obj files, which contain the city’s 3D map, and processes the triangles between source and destination in the engine. The list of tracked pathways is the end result. The first tuple position denotes the route types of direct, diffracted, and reflected light. Path Loss must be calculated. The tracer determines the overall route loss based on theoretical propagation models as the potential pathways are traced from the motor. The scene’s material is considered concrete in our work.

The calculation of the least Aggregate Path Loss, as illustrated in Equation (2) [22], is a crucial aspect of choosing the optimum route. Path loss is an attenuation that occurs as an electromagnetic wave travels through a channel, reducing its power density. Path loss can take several forms, ranging from natural radio wave propagation to diffraction path loss caused by interference, to saturation path loss caused by the presence of a signal that is not transparent to electromagnetic waves [23]. Between communication hops, we use the sum of all path losses. The wavelength of free space is defined as the ratio between the speed of light in meters per second and the carrier frequency in Hz, as indicated in Equations (2) and (3). The work [24] is the foundation for the equations.

(2) $$PathLoss=\sum_{0}^{e}\left(\frac{10.log_{10}(16.\pi .r^{2}.D_{e}^{2}}{\lambda^{2}}\right)$$

where;

(3) $$\lambda =\frac{c}{f_{c}}$$

This is how the RSSI is calculated in Equation (4). Were the communication devices can act as both an access point (AP) and a wireless client/station (STA) simultaneously. With this setup, you may use a single wireless device to build an AP that acts as a “wireless repeater” for an existing network. The AP-STA and STA-AP channels are assumed to be reciprocal. As a result, in both directions, PathLoss plus ShadowLoss is the same.

(4) RSSI=Tx_Power−(PathLoss+ShadowLoss)

### Simulation

We employ computer-based simulations to test and confirm our scientific results and evaluate the efficacy of the technique suggested in this paper since they are more adaptable and low-cost when compared with real-world implementation. Because both the *OpenAI Gym* [18] tool and the Python programming language are computationally efficient and useful for simulation, we integrate both.

For this simulation, we established a one-hour time constraint and used Urban Mobility Simulation (SUMO) [25] to generate a realistic scenario. The open-source SUMO software allows for the simulation of land transport modes. It creates a network simulation using autos as nodes. It is a collection of technologies that work together to create the scenario we employed in our experiment [26].

**Figure 2:**
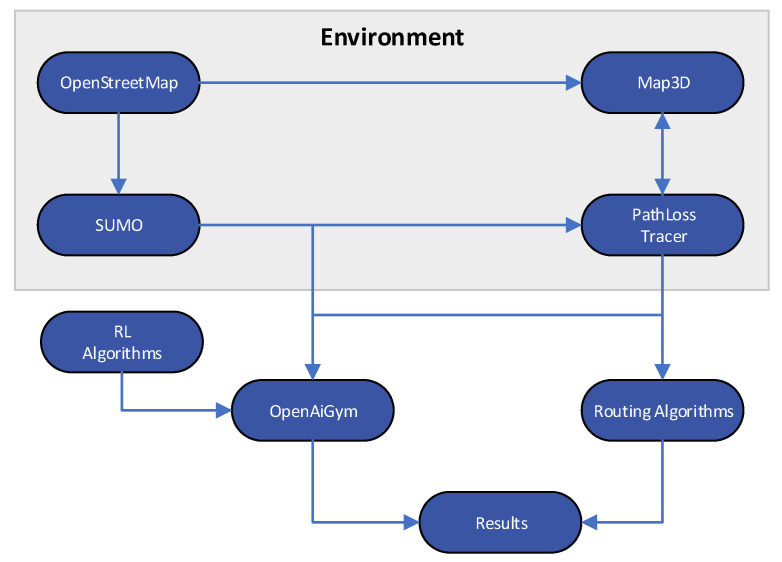
Simulation diagram.

Figure 2 depicts the configuration project for our simulation environment, which requires multiple stages to complete. To begin, we import the city map into the *OpenStreetMap* application. This map was used by *Sumo* to replicate random automobile journeys, and by *Map3D* to create a representation of the city. The output of *Sumo* and the *Map3D* feed the proposed *OpenAI Gym* algorithm and the other two routing algorithm implementations used in current VANETs, the *DSR* and *DSDV* routing protocols.

**Figure 3:**
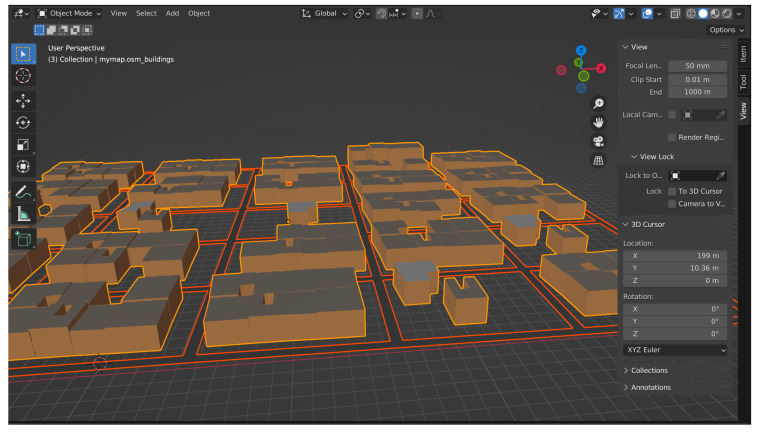
Three-dimensional map of the city.

Figure 3 shows a city with a three-dimensional grid layout. This model uses a grid road design to mimic the movement pattern of mobile nodes in metropolitan situations. Horizontal and vertical streets are included in this mobility model, as well as randomly positioned nodes on the map at the start of the simulation and the ability to change lanes [27]. In our proposed model, the third dimension is important, to better calculate the *RSSI* and *Path Loss* due to interference from urban buildings and constructions.

## 4. Results and Discussion

The DSR and DSDV procedures, as well as our suggested reinforcement learning method, were compared. As mentioned in the simulation chapter, each protocol was tested using evenly dispersed cars and random displacement to produce traffic. The suggested methods were also put to the test using three distinct communication paths: direct path, diffracted path, and reflected path.

The following measures were utilized to assess the suggested protocol’s effectiveness: The average path lifespan, that is, the path that connects the same source and destination. During the simulation time, the number of connection transitions, or route alterations between the source and destination paths, was counted. And there’s the average path length, which is the amount of hops the routes have on average.

Six different simulations were compared: GPPO: uses the RL PPO algorithm and is fed with pathLoss. DPPO: uses the RL PPO algorithm, but only takes into account the distance between vehicles. GA2C: uses the RL A2C algorithm, fed with pathLoss. DA2C: A2C algorithm, with vehicle distance only. DSDV and DSR: Legacy network routing algorithms, are still used nowadays.

**Figure 4:**
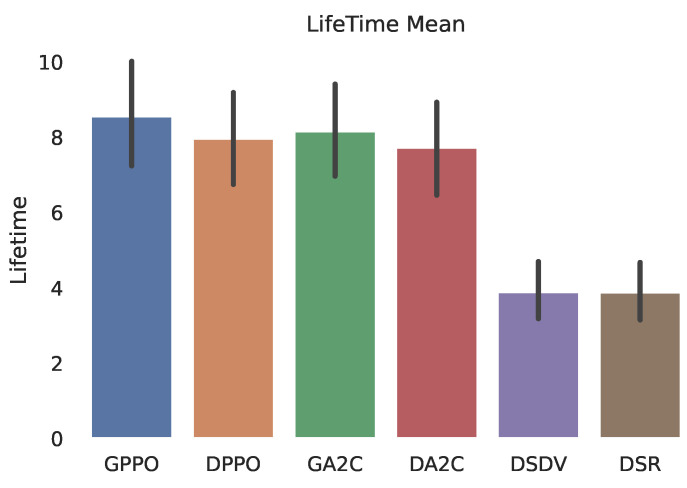
Path life time.

As shown in Figure 4, PPO and A2C agents obtain a higher normalized average lifetime of the path, than standard DSR and DSDV communication protocols. In other words, the route paths chosen by reinforcement learning agents have a longer life span. The confidence interval of the obtained data can also be seen. The difference between the path chosen by the PPO agent compared to DSR or DSDV algorithms doubles the lifetime of the route path on average, during the simulation time.

**Figure 5:**
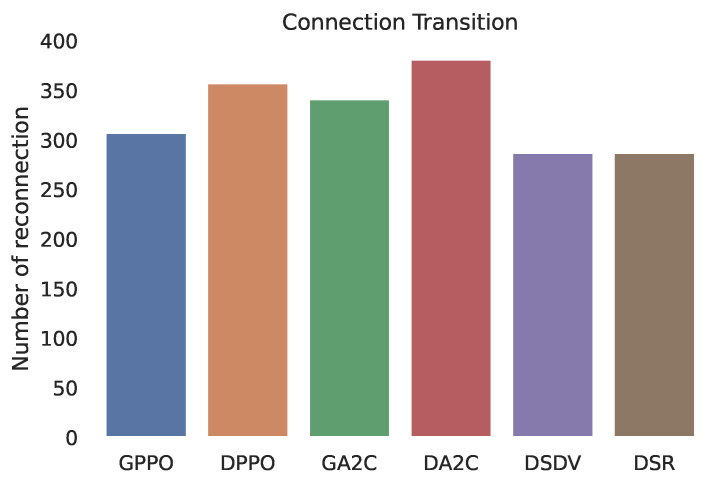
Number of reconnections.

This evaluation is critical in data communication, especially in mobile vehicular networks, increasing the route’s lifetime, consequently decreasing the number of reconnections and overheads of the protocol, as shown in Figure 5. We achieved fewer connection transitions with the proposed methodology GPPO and GA2C than DPPO and DA2C and DSR or DSDV. It means that we can have less or even the same amount of reconnections, but with a longer lifetime route.

**Figure 6:**
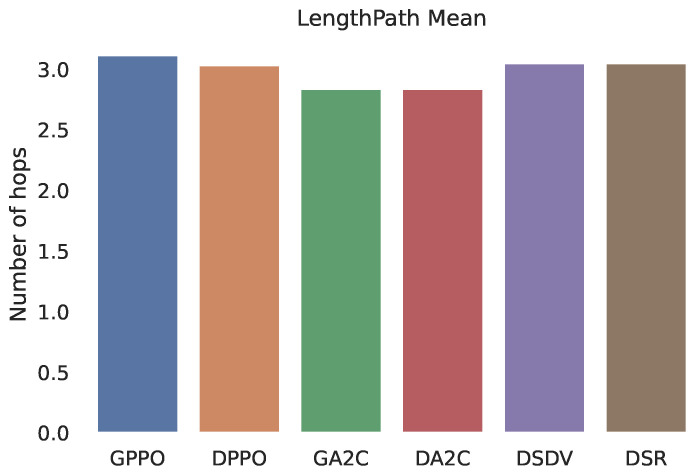
Hop number of the chosen path.

Although both artificial intelligence agents are superior to conventional protocols in all the simulations carried out, the PPO agent had a slight advantage compared to the A2C agent, either in the lifetime of the path or in the number of transitions. As the lifetime of the chosen route paths is significantly longer, this does not mean that the selected route is the shortest; that is, it has fewer hops. As seen in Figure 6, the paths chosen by intelligent agents have an equal or more significant number of leaps, as expected. Even if the number of hops is greater than the short path algorithm, the difference is not significantly more prominent.

Two other figures were used with the same metric but exposed differently to visualize the lifespan gain compared to the path length. Data normalization, which is a method for structuring data attributes to improve the cohesion of entity types within a data model, was used. Our goal is to reduce data duplication, which is crucial for visualizing the differences in behavior between the protocols.

**Figure 7:**
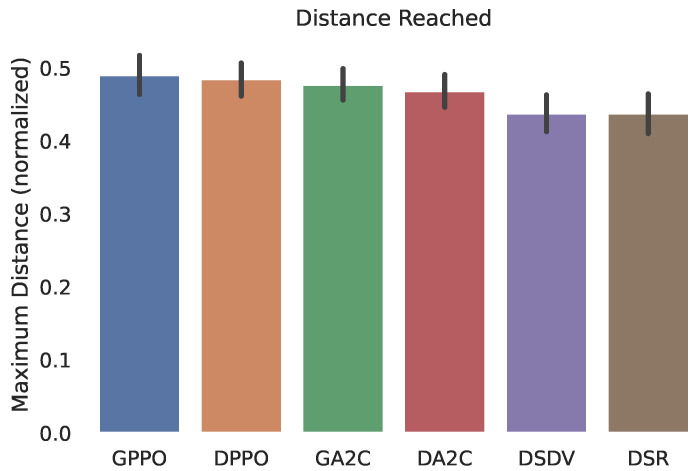
Normalized maximum distance reach of the path.

In Figure 7, we can see that our proposed algorithm achieved greater communication distance (measured in meters), compared to the other protocols. This was not an expected feature, but it was recognized in the results. The confidence interval of the obtained data is also shown.

**Figure 8:**
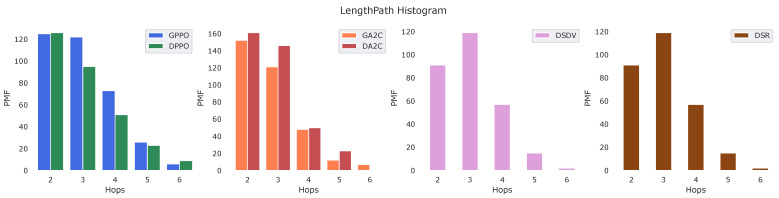
Histogram length of the path.

**Figure 9:**
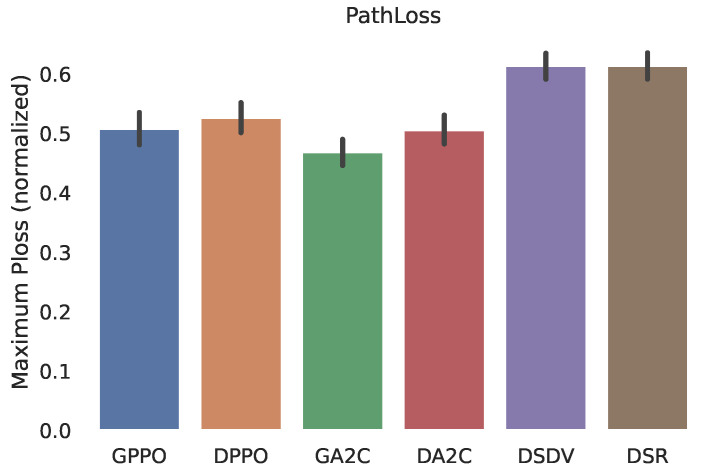
Maximum Path Loss normalized.

On the other hand, the Figure 8 shows the Probability Mass Function (PMF) of the width of the path length measured in hops. Here we can see the difference in behavior between the algorithms. Another observed consequence of the application of our developed algorithm, it was possible to measure a lower level of signal loss of the chosen path, as can be seen in Figure 9. The data’s confidence interval is displayed.

## 5. Conclusions

We discussed the environment design process and showed how to use an OpenAI Gym environment to research VANET network routing algorithms. As a starting point for future exploration and research, we implemented a reactive agent, a standard process, and an agent trained in PPO and A2C. The agent obtained higher reward strategies, implying that reinforcement learning agents can recognize different route paths. PathLoss can be reduced, data can be sent over longer distances, and the number of connections between source and destination can be reduced using intelligent learning algorithms that apply reinforcement learning. It was also possible to make the chosen path last longer by choosing one with a longer lifespan. That is, as compared to other traditional algorithms in use today, such as DSDC and DSR, they greatly improved the metrics analyzed. Complex networks with high mobility and specialized requirements, such as VANETs, should not be bound by traditional routing algorithms, but rather expand their horizons. An alternative is to adapt to the future of data transfer by using new techniques.

## Figures and Tables

**Table 1:** RSSI table.

Signal Strength	Meaning
<−67 dBm	Very Good
<−70 dBm	Good
>−70 dBm	Not Good
